# An Approach to Identify HLA Class II Immunogenic Epitopes in the Greek Population through Machine Learning Algorithms

**DOI:** 10.3390/jcm11237046

**Published:** 2022-11-29

**Authors:** Asimina Fylaktou, Georgios Lioulios, Katerina Tarassi, Alexandra Siorenta, George Ch Petasis, Demetris Gerogiannis, Ioannis Theodorou, Aliki G. Iniotaki, Angeliki G. Vittoraki

**Affiliations:** 1 National Peripheral Histocompatibility Center, Immunology Department, Hippokration General Hospital, 54642 Thessaloniki, Greece; 2 Immunology-Histocompatibility Department, “Evangelismos” General Hospital, 10676 Athens, Greece; 3 Immunology Department & National Tissue Typing Center, General Hospital of Athens “G. Gennimatas”, 11527 Athens, Greece; 4 Department of Computer Science & Engineering, University of Ioannina, 45110 Ioannina, Greece; 5 Laboratoire d’Immunologie, Hôpital Robert Debre, 75010 Paris, France

**Keywords:** machine learning, epitope matching, eplets, anti-HLA class II responses, alloimmune response, transplantation

## Abstract

Current pre-transplantation routine matching involves serum anti-HLA antibodies quantification but cannot always preclude unfavorable graft outcomes. Epitope-based matching is proposed as a more precise approach, but to date no epitope-matching algorithm provides a satisfactory predictive tool for transplantation outcomes. In this study, anti-HLA-II loci responses from 1748 patients were analyzed with unsupervised machine learning algorithms, namely principal component analysis (PCA) and antigenic distances, projected as dendrograms. PCA for anti-HLA-DR anti-bodies revealed three main clusters of responses: anti-HLA-DR51 combined with anti-HLA-DRB1*01, anti-HLA-DR52 combined with anti-HLA-DRB1*08 and anti-HLA-DR53 combined with anti-HLA-DRB1*10. The dendrogram for anti-HLA-DR confirmed the pattern and showed further bisection of each cluster. Common epitopes present exclusively in all HLA molecules of each cluster were determined following the HLA epitope registry. Thus, we propose that 19 out of 123 HLA-DR epitopes are those that mainly lead anti-HLA-DR responses in the studied population. Likewise, we identified 22 out of 83 epitopes responsible for anti-HLA-DQ and 13 out of 62 responsible for anti-HLA-DP responses. Interpretation of these results may elucidate mechanisms of interlocus cross-reactivity, providing an alternative way of estimating the significance of each epitope in a population and thus suggesting a novel strategy towards optimal donor selection.

## 1. Introduction

A fundamental principle in immunology is the response of the immune system to anything not recognized as self. In the setting of organ transplantation, human genetic diversity is the basis of the alloimmune response to foreign antigens expressed on the graft endothelial cells. The most important polymorphic antigens of clinical transplantation are the human leukocyte antigens (HLA). Gene-coding HLA molecules are expressed as polypeptide chains and are grouped according to their structure into class I (HLA-A, -B, -C) and class II (HLA-DR, -DQ, -DP) antigens. The differences (polymorphisms) among antigens of the HLA system derive from nucleotide substitutions expressed as different amino acids in the polypeptide chain. Evolution has led to a great level of polymorphism of the corresponding genes to such a degree that complete identity between two individuals is scarce. However, an adequate HLA match, especially in the DR locus, is crucial to a favorable outcome in solid organ transplantation [1,2].

HLA class II molecules are expressed mainly on antigen-presenting cells (B lymphocytes, dendritic cells and macrophages) and activated T lymphocytes and have also been found on normal microvascular endothelial cells of humans but not animals [3,4]. They are encoded by nine genes, DRA, DRB1, DRB3, DRB4, DRB5, DPA1, DPB1, DQA1 and DQB1. Each HLA class II molecule consists of a heterodimeric protein, comprising two transmembrane chains, α and β. Although the DR α chain gene exhibits greater variability than previously considered [5], nevertheless the peptide binding part of this chain is invariable and therefore anti-DR specific reactions are solely explained by polymorphisms of the β chain. Most of the DRB1 alleles are co-inherited with specific DRB3, DRB4 and DRB5 alleles. The DPA1 and DQA1 gene families also express great variability. However, the immunogenicity of each chain is deemed to be different, as mainly DQ β chains have been considered to be responsible for the anti-HLA-DQ alloreactions, whereas the formation of anti-HLA-DP alloantibodies is due to reaction against mainly DP α chains [6]. Nonetheless, a more modern consideration conceives epitopes as formed within the total α-chain/β-chain complex, not in an isolated amino chain only. Hence, anti-HLA-DQ antibodies are believed to recognize a 3D area formed by the intact molecule [7], a conception also potentially valid for anti-HLA-DR and DP antibodies.

Development of de novo HLA class II Donor Specific Antibodies (DSAs) has detrimental effects on graft outcome, as up to 40% of transplanted patients will present antibody-mediated rejection and graft dysfunction 5 years after DSA development [8]. In allotransplantation, inflammatory cytokines amplify HLA-DR and HLA-DQ expression on the activated graft endothelium after antigen–antibody binding. Graft endothelium, acting as a semi-professional antigen presenting cell, promotes inflammatory Th1 and Th17 CD4 memory alloreactivity on one hand and anti-inflammatory regulatory CD4 T cell (Treg) activation on the other hand; both reactions being on a dynamic balance. Several studies have shown that the presence of anti-HLA-DR and DQ antibodies mitigate a Treg response and promote inflammatory Th17 reactions, with detrimental effects on graft survival [9,10,11].

Donor-specific antibodies bind (recognize) polymorphic regions on the surface of HLA molecules, which are characterized as B cell epitopes. Each HLA molecule expresses a group of epitopes. As a rule, many HLA alleles share common epitopes. This cross-reactivity results in a broader humoral response than the one expected by the donor’s phenotype. Of note, these cross reactions among HLA class II are mainly intralocus and do not occur among different loci, as happens with HLA class I antigens. For this reason, the prediction of immune responses against specific HLAs following organ transplantation is of utmost importance for optimal donor selection [8].

Availability of the Single Antigen-Coated Beads (SAB) method and analysis with multi-color flow cytometers (Luminex) allows the detection of anti-HLA antibodies directed against each molecular complex present on the beads. The SAB method is capable of studying the epitope recognition range of serum antibodies against the “foreign” graft epitopes of the population. The data emerging from this method are of great complexity, rendering the recognition of humoral patterns challenging but also precise, thus adding new insights to the anti-HLA response.

In a recent study, we examined patterns of anti-HLA class I antibodies in 1066 Greek patients, either transplanted or on the waiting list, and evaluated antigenic distances among HLA alleles with the aid of simple machine learning algorithms [12]. In an older study, we searched patterns of interlocus anti-HLA class II immune response patterns in a large Greek population [13]. The commonplace factor of these studies was the use of dimensionality reduction algorithms, aiming at an unsupervised analysis of anti-HLA immune responses. Probably, the most useful findings of these studies were that the antigenic distances among different HLA antigens could be defined experimentally at a population level. To a certain degree, these findings reflect epitopic targets, which are commonly co-recognized in the studied population.

The purpose of the present study is to evaluate anti-HLA class II humoral response patterns according to known HLA B cell epitopes and to highlight the eplets that are co-recognized in this population. To this aim, we applied simple unsupervised dimensionality reduction algorithms to a high-dimensional dataset of anti-HLA class II antibody measurements from the three histocompatibility centers of Greece that support organ transplantation.

## 2. Materials and Methods

### 2.1. Patients

In the present study, we analyzed sera of 1748 patients tested for anti-HLA class II antibodies, pre- and post-transplantation. Patients had either received a renal graft or were on a waiting list for renal transplantation and were followed up in one of the three major histocompatibility laboratories of Greece. Transplanted patients were examined for DSAs annually, while patients waiting for transplantation were examined every three months, according to the Greek protocol. One sample of each patient was analyzed. In case of multiple sera, only the most recent was included in the study.

The study protocol was approved by the Health Research and Ethical Board of “G. Gennimatas” Hospital of Athens, “Hippokration” Hospital of Thessaloniki and “Evangelismos” Hospital of Athens. Informed consent for the study was obtained from all patients prior to the analysis.

### 2.2. Anti-HLA Antibodies Measurement

Sera from patients with known anti-HLA reactivity, or after a positive LSM screening test, were analyzed with the SAB test on a Luminex 100 flow multicolor cytometer to estimate the raw Mean Fluorescence Intensities (MFI) of anti-HLA class II antibodies. In addition, in cases of clinical suspicion of rejection, SAB tests were performed even with a negative LSM screening test. Beads for detection of anti-HLA-DR responses were coated with a single protein, whereas beads for detection of anti-HLA-DQ and anti-HLA-DP responses were coated with two proteins, one for chain A1 and one for chain Β1. The tests were performed according to the manufacturer’s instructions and under the same protocol in all three laboratories. Sera were stored at −20 °C until analysis. EDTA pretreatment was performed on all sera to prevent the prozone effect [14].

### 2.3. Descriptive Statistics

Raw MFIs from each patient were exported from the Fusion Analysis software database for each individual bead and used as input for dimensionality reduction by Principal Component Analysis (PCA) [15]. In order to retain the unsupervised character of PCA, we included all MFI values produced by the device, irrespective of being considered positive or negative. A separate PCA analysis was performed for each of the major HLA class II loci, HLA-DR, HLA-DQ and HLA-DP. Before PCA analysis data were centered and scaled to unit, PCAs were performed in R with the FactoMiner and Factoextra packages [16].

We also performed a second analysis based on hierarchical clustering for each locus separately and projected the data on a two-dimensional plane as phylogenetic trees or dendrograms [17]. Before analysis, the raw MFI values were centered and scaled to unit. These data were used as feature vectors and their Minkowski distances were used for a Wald’s-based bottom to top hierarchical clustering. Minkowski distances were preferred to pairwise Spearman’s linear correlations, in order to capture potential non-linear correlations [18].

In order to explain the formation of clusters of HLA class II alleles, we defined common epitopes present exclusively in each of the formed clusters. Information on epitopes present in each allele, as well as the ElliPro scores, came from the HLA Epitope Registry [https://epregistry.com.br/ (accessed on 1 October 2022)], version 3.0, DRB, DQ, DP databases, Luminex alleles [19].

## 3. Results

### 3.1. Clustering and Epitopic Analysis of Anti-HLA-DR Immune Response

**Figure 1:**
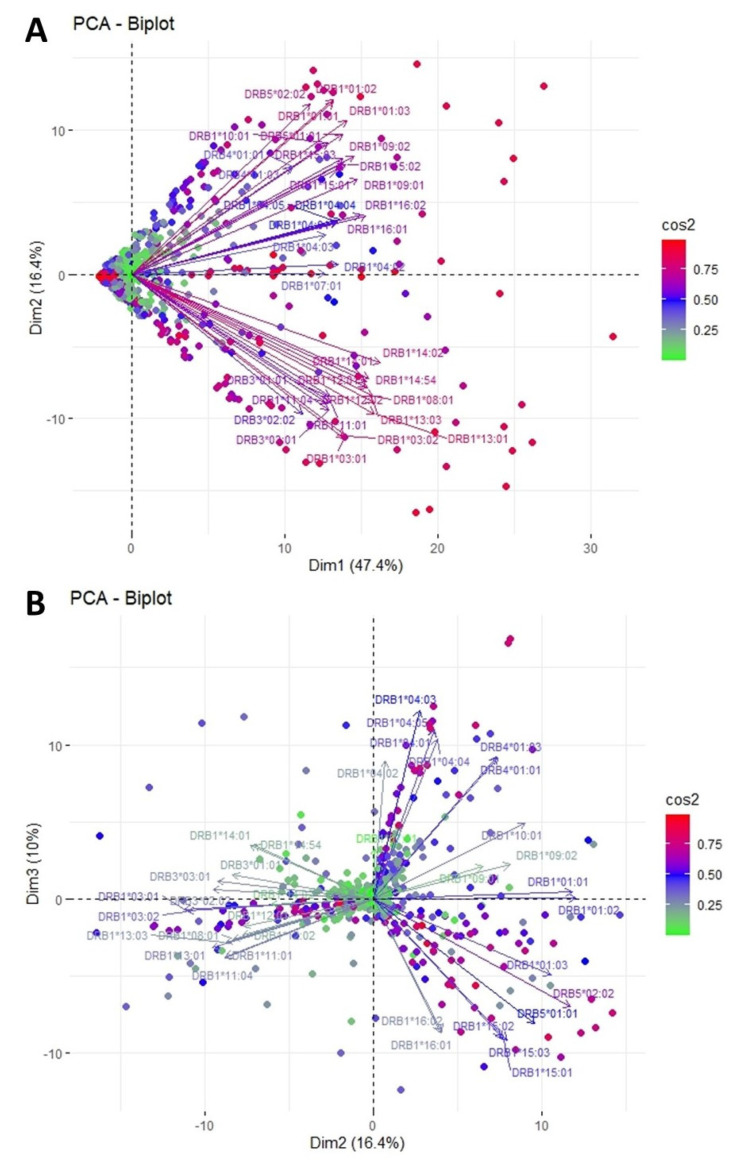
PCA biplot projections of anti-HLA-DR responses. (**A**) Projection of the first and second eigenvectors of the covariance matrix. (**B**) Projection of the second and third eigenvectors of the covariance matrix. Each arrow represents a specific anti-HLA-DR reaction, while each point indicates individual reactions. Colors of loadings and points represent the cos2 value of the explained variance for variables and individuals, respectively.

Figure 1 shows PCA biplot projections for the responses against HLA-DR alleles. Arrows (loadings) represent the different alleles, while each dot represents an individual response. The color and length of each loading is proportional to the corresponding explained variance. The defined plane of the first two eigenvectors of this PCA is shown in Figure 1A. The projections of the data in this plane explain 63.8% of the total variance. In this figure, two well-defined clusters of variables are observed. The first cluster pointing to the upper right quadrant includes long loadings representing anti-HLA-DRB1*01, *09, *10, *15 and *16, as well as HLA-DRB5 (DR51) reactions. Responses against HLA-DRB1*03, *08, *11, *12, *13 and *14, as well as HLA-DRB3*03 (DR52), form a second cluster roughly orthogonal to the former, which directs to the lower right quadrant of the plane. The similar length and direction of the arrows in each of the groups imply a strong correlation of the corresponding responses in the majority of the patients, while the orthogonality between the clusters suggests that these responses occur independently in the studied population. Of note, a less distinct third cluster forms with direction similar to the first described cluster but with evidently shorter loadings. This cluster includes responses against DRB4 (DR53) and DRB1*04 alleles. Finally, the response against DRB1*07 lies independently on the horizontal axis with direction to the right.

Figure 1B depicts the plane defined by the projection of the second and third eigenvector. This plot adds 10% more to the variance explained by this PCA and points to a “rarer” phenotype. On this plane, anti-HLA responses form three main beams of arrows, each with distinct direction. The cluster occupying the lower right quadrant consists mainly of anti-HLA-DRB1*15 and *16 along with DRB5, which were also tightly clustered in the plot of the first and second eigenvector. However, while anti-HLAs DRB1*01, *09 and *10 were associated with anti-HLA-DRB1*15, *16 and DRB5 in the first projection, this time, their corresponding loadings appear almost perpendicular, implying that to a small extent these responses can be independent across the population. On the contrary, the second beam of the first projection, embodying anti-HLA-DRB1*03, *08, *11, *12, *13, *14 and HLA-DRB3*03, remains grouped in this projection too, suggesting a strong correlation among these responses. Additionally, on this projection a third cluster can be observed, formed by long loadings representing anti-HLA-DRB1*04 and DRB4. These variables that, although clustered with HLA-DRB1*01, *09, *10, *15 and *16, were explained weakly on the first projection (relatively short arrows), now point to the upper right quadrant and appear not to associate with other responses. Response against DRB1*07 is weakly represented in this projection too and not clustered along with other groups.

**Figure 2:**
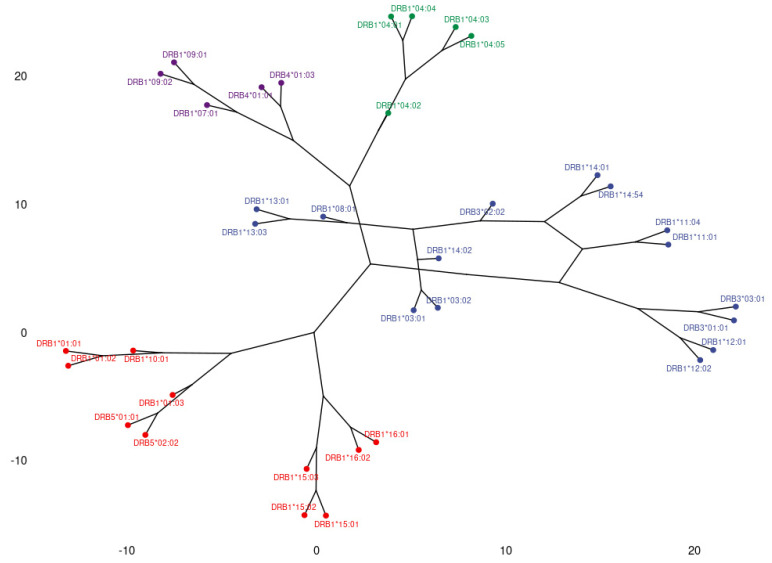
Dendrogram representing anti-HLA-DR response correlations, based on Minkowski distances of antigen specific feature vectors. Three main branches corresponding to distinct groups of responses are observed. Group A, in blue, includes responses against DRB1*03, *11, *12, *13, *14 and DRB3 (DR52), as well as anti-DRB1*08 response, group B (in green and purple) represents responses against DRB1*04, *07, *09 and DRB4 (DR53) and group C (in red) includes responses against DRB1*15, *16 and DRB5 (DR51), as well as anti-DRB1*01 and *10 responses.

Consequently, antigenic distances were calculated by using the Minkowski metric and the results were depicted in a phylogenetic tree. Figure 2 depicts the dendrogram of the phylogenetic relationship of immune responses against HLA-DR alleles. In general, the main branching of the tree follows clustering as emerged from the corresponding PCA. However, secondary branching reveals some interesting associations. For example, whereas the right-pointing branch in Figure 2 (group A) includes all components of DR52 responses, the secondary branching uncovers a tighter association among anti-DRB3*01 and *03, as well as anti-DRB1*12 responses, which tend to occur independently from DRB3*02 and DRB1*03, *11, *13 and *14, alloreactions that form a separate secondary branch. Moreover, the “odd” participant DRB1*08, which is classically not included in the DR52 serotype, is incorporated in the latter cluster, indicating a tendency to co-occur mostly with anti-DRB1*13 but also with anti-DRB3*02 and DRB1*03, *11 and *14. In a similar way, anti-DRB1*04 response forms a separate secondary branch within the DR53 main branch (Group B, pointing upwards in Figure 2). In this case, anti-DRB4 responses appear to accompany mainly anti-DRB1*07 and *09 and not anti-DRB1*04, at least in a part of the population studied. Finally, as seen in the downward-pointing branch, anti-DRB1*01 and *10 responses, not included in the DR51 serotype, appear more closely associated with anti-DRB5 than anti-DRB1*15 and *16 do. The significance of these observations remains to be investigated. 

In the last step of our approach, we suggest an explanation for the forming of the anti-HLA response groups by locating common epitopes present within each group obtained from PCAs and phylogenetic analysis. We assume that each of the described groups reflects the tendency of antibodies to recognize specific “shared” epitopes among HLA alleles. 

According to the HLA-DR epitope registry, HLA-DR expresses in the β polymorphic chain a total of 123 epitopes, of which 117 are “shared” between different DR alleles and only 6 “private” epitopes are restricted to one DR Luminex allele. As shown in Table 1, only 32 of 123 (26%) DRB epitopes are restricted in only one of the PCAs groups; out of them, 8 (25%) are present in only one of the alleles tested in the study, while 5 additional epitopes have been characterized by a very low or a low Ellipro Score. The remaining 19 epitopes (30C, 30G, 37S, 71A, 96EV, 108T, 142M, 48Q, 96Y, 11STS, 31FH, 37L, 57A, 57DE, 70QQ, 74R, 77N, 96HK and 98Q) appear to drive anti-HLA-DR responses in the studied population.

### 3.2. Clustering and Epitopic Analysis of Anti-HLA-DQ Immune Response

**Figure 3:**
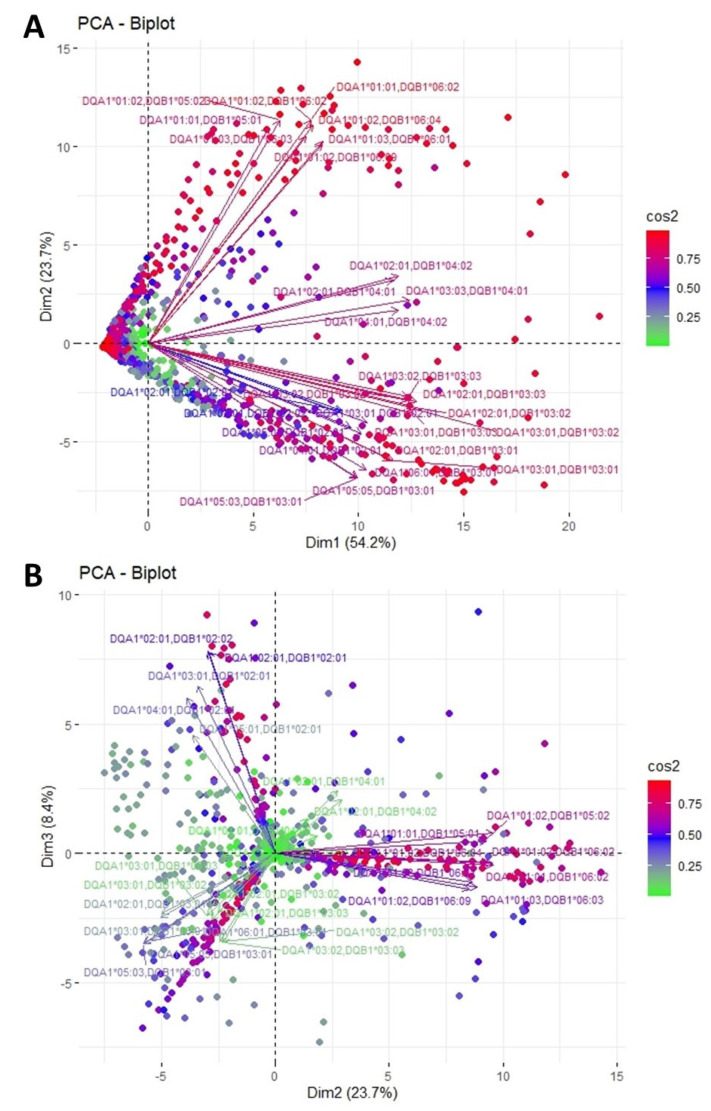
PCA biplot projections of anti-HLA-DQ responses. (**A**) Projection of the first and second eigenvectors of the covariance matrix. (**B**) Projection of the second and third eigenvectors of the covariance matrix. Each arrow represents a specific anti-HLA-DR reaction, while each point indicates individual reactions. Colors of loadings and points represent the cos2 value of the explained variance for variables and individuals, respectively.

Figure 3 shows an analogous PCA projection of the responses against HLA-DQ epitopes. The plane defined by the first and second eigenvectors is shown in Figure 3A and explains a rather satisfactory 77.7% of the total variance. On this plane, variables form three solid groups of loadings, with limited angular divergence among arrows within each group, suggesting a tendency of the corresponding responses to occur concurrently. More specifically, the upper cluster consists of anti-HLA-DQB1*05 and DQB1*06, all of which are represented by long arrows with similar lengths and direction, indicating a clear association among these variables. The lower cluster includes responses against HLA-DQB1*03, along with DQB1*02 depicted by relatively shorter arrows. The whole cluster directs perpendicularly to the upper group, implying independent occurrence of the corresponding responses. Finally, in between these two first clusters lies a third one shaped by DQB1*04 responses. It is worth noting that anti-HLA-DQA1 responses were scattered within the clusters, indicating that responses against DQ alleles are driven mainly by β chain.

To reinforce our observations, we analyzed the second and third eigenvector delimited plane, which explains an additional 8.4% of the total variance, raising the total variance explanation to 86.1% (Figure 3B). In this second plot, anti-HLA-DQB1*05 and DQB1*06 appear colinear, occupying the right part of the plane. The corresponding arrows remain long with a minimum divergence, showing the strong correlation of these responses within the studied population. On the left side of the plot lay responses against HLA-DQB1*02 and *03, forming two discreet groups orthogonal to each other. Whereas these responses were colinear on the first plot, the differences in arrow length imply that the responses do not occur in parallel. This concept is confirmed by this second plot. Finally, anti-DQB1*04 responses form a fourth distinct cluster, although with rather short loadings, indicating a modest contribution to the variance explained. Once more, anti-HLA-DQA1 responses were scattered within the clusters, indicating the higher immunogenicity of β chain.

**Figure 4:**
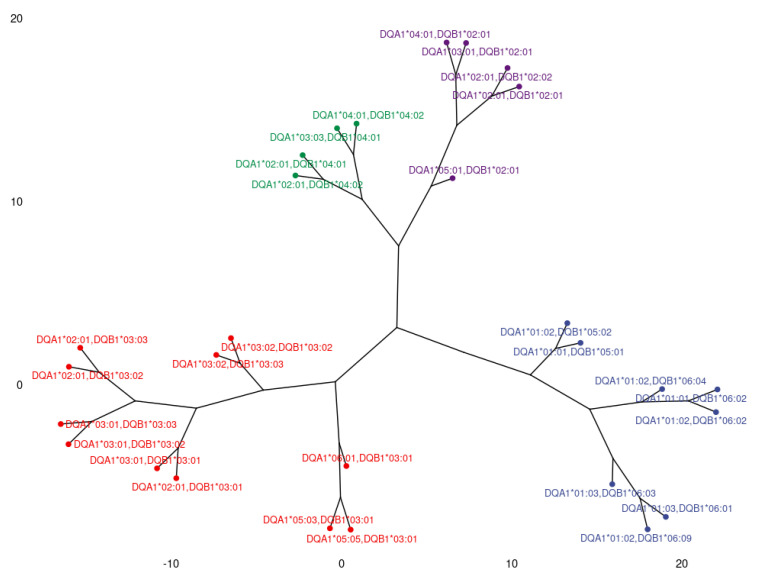
Dendrogram representing anti-HLA-DQ response correlations based on Minkowski distances of antigen specific feature vectors. Three main branches corresponding to distinct groups of responses are observed. Responses against distinct β chains gather within a separate group, while responses against distinct α chains appear in all groups. Group A, in blue, includes responses against DQB1*05 and *06 as well as DQA1*01, group B (in green and purple) represents responses against DQB1*02 and *04, and group C (in red) includes responses against DQB1*03.

Figure 4 depicts the phylogenetic associations of anti-DQ responses. As shown by the corresponding PCA, the anti-DQ response appears to be led mainly by β1 chain, as there is a clear clustering of anti-DQB1*05 and *06 responses into one main branch and a separate main branch that includes anti-DQB1*03 responses. However, contrary to PCA, in this approach, anti-DQB1*02 and *04 responses combine in the same main branch.

Similarly, HLA-DQ locus expresses in α and β polymorphic chains 83 epitopes, 5 of which appear only in one allele. Table 2 shows 28 (33.7%) epitopes present exclusively in one of the clusters formed by PCA. We exclude the five epitopes present in only one allele, as well as one epitope with a low Ellipro score. The remaining 20 epitopes present in the B1 chain (30H, 37YV, 52PQ, 55RPD, 57V, 67VG, 70GT, 86A, 87F, 87Y, 116I, 130Q, 56L, 55PP and 52LL) as well as 2 epitopes of A1 chain (52SK and 129QS) are potentially those that drive immune responses in the examined population.

### 3.3. Clustering and Epitopic Analysis of Anti-HLA-DP Immune Response

**Figure 5:**
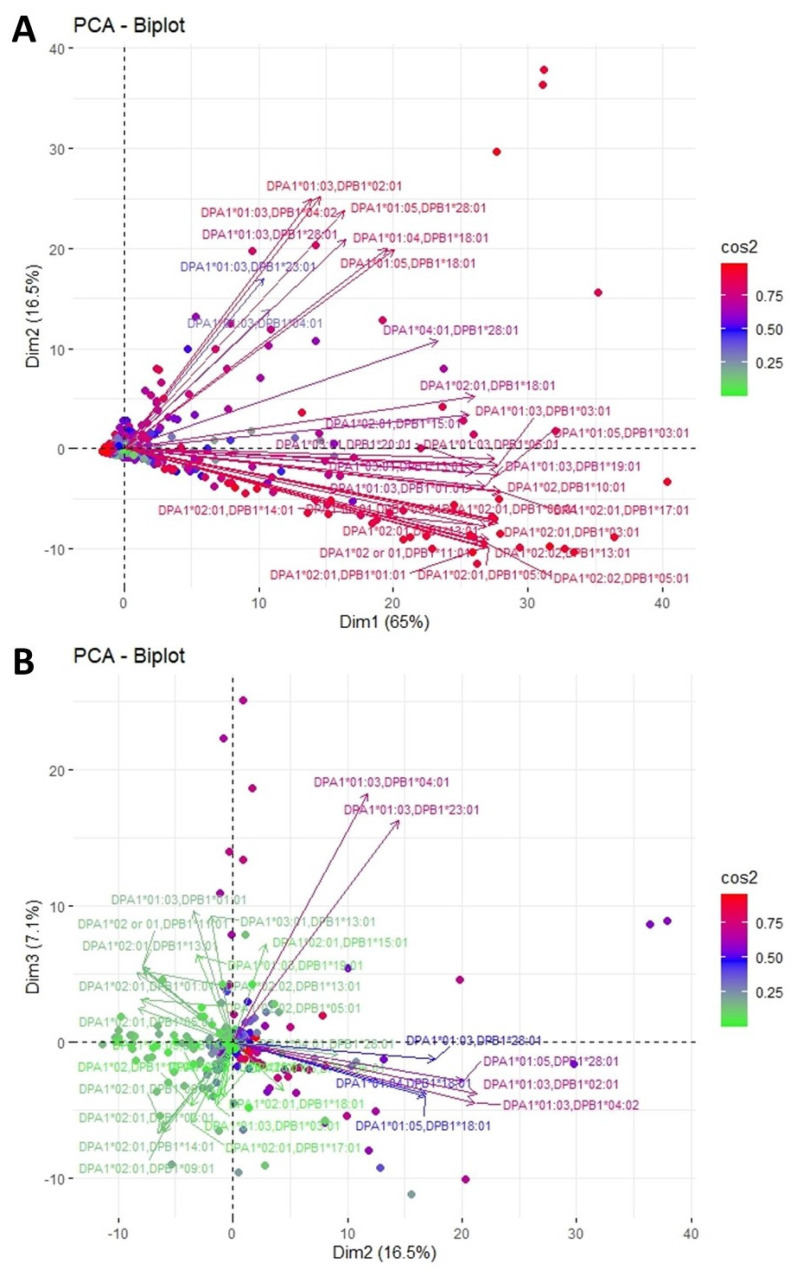
PCA biplot projections of anti-HLA-DP responses. (**A**) Projection of the first and second eigenvectors of the covariance matrix. (**B**) Projection of the second and third eigenvectors of the covariance matrix. Each arrow represents a specific anti-HLA-DR reaction, while each point indicates individual reactions. Colors of loadings and points represent the cos2 value of the explained variance for variables and individuals, respectively.

Figure 5 shows a third PCA projection depicting the plane defined by the first two eigenvectors of the anti-HLA-DP responses. This analysis captures 81.5% of the total variance. Two main clusters of loading are observed, the former occupying the upper right quadrant and the latter the lower right part of the plane. The upper beam consists largely of anti-DPA1*01 in combination with anti-DPB1*02, *04, *18, *23 and *28, whereas the lower beam includes the total of anti-DPA1*02 responses in combination with anti-DPB1*01, *03, *05, *06, *10, *11, *13, *14 and *17. Finally, in this particular plot, there is a major gathering of all dots, each representing an individual response, by the axis origin, with only a few of them laying far apart on the plane. This finding indicates a moderate immune response against DP antigens in the studied population. Analyzing the second- and third-dimension plots, one observes that the lower cluster is further divided into two separate groups with opposite directions. However, the third dimension adds a mere 7% of variance explanation and the loadings of the corresponding variables are relatively short, showing their limited contribution to the total variance.

**Figure 6:**
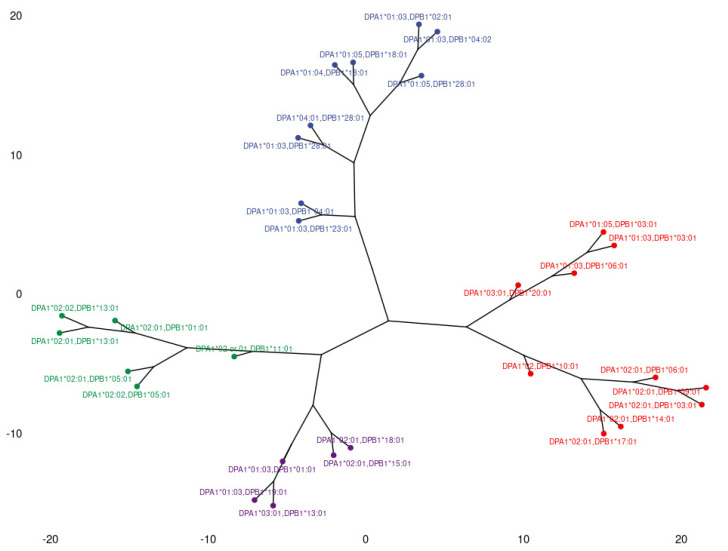
Dendrogram representing anti-HLA-DP response correlations, based on Minkowski distances of antigen specific feature vectors. Three main branches corresponding to distinct groups of responses are observed.

The phylogenetic analysis of anti-HLA-DP responses is shown in Figure 6. This dendrogram consists of three branches. The upward-directing branch corresponds to the loadings that form the upper cluster of the DP PCA. The two lower-side-directing branches correspond to the lower cluster of the DP PCA, as it was split by the second–third dimension plot. By examining together the PCAs and the dendrogram, one could conclude that these lower responses tend to occur together in a great proportion of the population but can also tend to one direction or the other in some patients, as shown by the two branches of the dendrogram.

Finally, following the same steps as the anti-HLA-DP responses, we conclude that 10 out of 45 epitopes present in the DPB1 chain (84GGPM, 33EYA, 35YA, 55EAE, 57D, 69R, 76I, 76V and 84DAEV) and 3 out of 17 in the DPA1 chain (50R, 127P and160V) may drive anti-HLA-DP responses (Table 3).

## 4. Discussion

The present study provides a systematic approach to the human alloresponse against HLA class II antigens in the Greek population through simple machine learning algorithms. The main purpose of this study is to gain knowledge about the clustering of anti-HLA class II antigen production and how this clustering can be explained according to published or suggested epitopes of HLA class II alleles.

Several studies during the last years have proved the significance of anti-HLA class II antibodies in the setting of solid organ transplantation. Whereas HLA-DR molecules were deemed the main immunogenic factor to the production of anti-HLA antibodies, recently increased evidence also supports a major role of DQ and DP molecules [20].

HLA class II incompatibility poses a higher risk for post-transplantation DSA development compared with HLA class II compatible grafts [21]. However, it must be mentioned that not all the newly formed in the post-transplantation period anti-HLA antibodies are specific to donor antigens. In a study published in 2011, 176 out of 520 kidney transplanted recipients were positive for anti-HLA antibodies against third party HLA, (non-DSAs), the great majority of whom (132/176, 75%) were pre-transplant sensitized. DSAs and non-DSAs were found to be independent predictors for graft loss [21]. These findings could be explained by the presence of alloantibodies that recognize shared epitopes/eplets between the foreign graft HLA and third-party HLA. Nowadays, the interest on eplet matching is constantly increasing. The molecular typing of HLA molecules and a deeper apprehension of their 3D structure has led research interest to the effects of amino acid substitutions among HLA molecules of the same family on graft outcomes. A first approach was attempted by identifying the absolute number of eplet mismatches and comparing outcomes between recipients with high- or low-mismatch loads. However, not all mismatches have the same impact or immunogenicity, with the potency of the alloresponse depending on the exact location and the properties of the corresponding amino acid. The Eurotransplant approach, on the other hand, is based on identifying acceptable mismatches in highly sensitized recipients by excluding eplets to which the patient has developed, or will possibly develop, alloantibodies. This concept has been in use for about three decades, reporting rather satisfactory graft survival [22]. More recently, based on molecular modeling of crystallized antigen–antibody complexes, Duquesnoy et al. determined potential antibody binding regions on HLA molecules and developed HLAMatchmaker, an algorithm that allows donor selection with minimum eplet mismatch load [23]. An additional algorithm, Predicted Indirectly ReCognizable HLA Epitopes (PIRCHE-II), predicts in silico the number of HLA mismatch-derived epitopes that can be presented to helper T cells [24]. Nonetheless, the up-to-date existing data are still poor and more research is needed before wider application is feasible [25].

In the current study, we analyzed the data of immune responses against HLA class II molecules of Greek patients who were either transplanted or on a waiting list for transplantation. The great complexity of the data studied renders the use of dimensionality reduction algorithms imperative. Therefore, we initiated our approach with PCA, a method first introduced more than a century ago but still in wide use [26]. We analyzed our data for each class II locus separately and we observed the formation of distinct clusters of loadings in all different loci analyzed. As each loading represents a distinct variable that is the response against a specific HLA molecule, we assume that responses that form a group of loadings tend to occur together, at least in a certain proportion of the population, and recognize a number of common molecular targets. In all three cases, the plot of the two first principal components captured a high-enough variance explained, indicating the tendencies of certain anti-HLA reactions to co-occur in the studied population. On top of this, the plot of the second and third principal components added an additional approximate 10% of the variance explained and showed the tendencies of anti-HLA reactions in a different minor portion of the population. Together, all three first dimensions explain more than 80% of total variance for HLA-DQ and HLA-DP loci, whereas this percentage is slightly lower (73.5%) for HLA-DR. However, the plane defined from the third and fourth PC did not give us any useful information for this dataset, despite providing an additional 7% to the explanation of the total variance. The corresponding eigenvalue scree plot and the PCA biplot of third and fourth PCs are seen in Supplementary Figures S1 and S2. The grouping of anti-HLA responses was performed with phylogenetic analysis with dendrograms too, that not only confirmed PCA results but also offered an insight into response allocations within each group. However, contrary to PCA that provides a multi-level aspect referring to different parts of the population in each dimension, phylogenetic analysis yields a more comprehensive overview of the correlations among the variables.

According to the HLA class II epitope registry, more that 80% of the recorded antibody targets (eplets) are locus specific, resulting in the production of intralocus antibodies as a rule. Epitopes found exclusively in one of the formed groups were identified as potentially highly immunogenic for this group of responses. In our analysis, we excluded those found in only one allele, as well as those with low or very low Ellipro scores [27].

In a recently published study, we performed a similar analysis, in which the grouping of responses against HLA class I molecules were featured and potential immunogenic epitopes were recognized, some of which have already been proved in the literature to date [12]. Moreover, an agnostic analysis of interlocus cross-reactivity was also presented, which, while giving a holistic depiction of anti-HLA responses in the Greek population, did not provide a suggested mechanism with implications in the clinical setting [13].

PCA plotting of anti-HLA-DR responses resulted in the formation of three main groups. It is of great interest that this model reveals some unexpected associations among anti-HLA responses. Antibodies against DRB1 alleles known not to relate to DRB3 (DR52), DRB4 (DR53) and DRB5 (DR51) alleles appear to have a strong correlation with anti-DRB3, DRB4 and DRB5 in both plots. Characteristically, the loading representing anti-DRB1*08:01 antibodies is collinear to DR52 representing loadings, whereas anti-DRB1*10:01 loading lays within the DR53 response representing loadings. Moreover, anti-DRB1*01:01, :02 and :03 are all but coincident to anti-DRB5 loadings. These findings indicate the presence of common epitopes between the corresponding alleles that could induce unexpected cross reactions. In the same analysis, we observe the presence of responses against DRB1*07 and *09 alleles. The rarity of this allele in the Greek population led us to the conclusion of either a strong response of these patients having “contact” with it or, more probably, the formation of these antibodies due to cross reaction. The dendrogram of anti-HLA-DR responses provided a similar grouping; however, it added some interesting information. The grouping of anti-DRB1*09 and *07 responses with anti-DRB4 indicates a potential cross-reactivity among these molecules, rather than with DRB1*04, which belongs to the same PCA group. A similar association might occur among anti-DRB1*01, *10 and anti-DRB5 responses.

The clustering of anti-HLA-DQ responses resulted in the formation of four groups. In three of these (including DQB1*02, *03 and *04 responses, respectively), responses appear to be driven by the DQβ chain, as all the epitopes identified as potentially immunogenic are found in DQβ chains. However, regarding the fourth group consisting of DQB1*05 and *06 responses, our method indicates that the DQα chain might also elaborate this group’s response, as eplets 52SK and 129QS are part of DQa chain. This comes in accordance with references that a minority of anti-HLA responses against DQ locus recognize the DQα chain or a combination of DQα and β chains [28]. Our approach indicates that such patients should belong only in a distinct group and should form antibodies against DQB1*05 and *06 chains in combination with DQA1*01. Of note, the difference of the clustering for the DQB1*04 and *02 groups, between PCA and the corresponding dendrogram, cannot be explained by eplet concurrence, as no common epitope was recognized exclusively in the group of these two.

The analysis of anti-HLA-DP responses highlighted the presence of one main cluster of responses (the upper one in the first and second dimension plot in the corresponding PCA, which predominates in the second and third dimension plot). Based on the rather limited contribution of the third dimension in PCA (7.1%), one can conclude that the majority of the population presenting an anti-HLA-DP response recognizes antigens belonging to this very group. Epitopes identified as immunogenic by our approach belong largely in DPβ chain, which seems to drive the immune response in this group. However, the low number of anti-HLA-DP responses in the studied population limits the validity of these results.

Our approach provides an alternative way to previously described methods towards an optimal eplet mismatch selection by determining the most immunogenic epitopes of HLA class II molecules and thus allowing a stratification of the clinical significance of each mismatch. Based on real world data, we hope that our results will contribute to the validation of current algorithms in the interest of patients.

A major limitation of this study is that anti-HLA responses were measured only in patients pre- or post-transplantation in a geographically constricted population of mainly Caucasian origin. This fact limits the universality of conclusions regarding the clustering of alloresponses and epitopic analysis, without, however, compromising the effectiveness of the method. Nonetheless, more studies are essential to validate our results, especially in populations of different origin. It should be mentioned that this is a part of an ongoing study, as data from the three histocompatibility laboratories are constantly collected. Data from the unsupervised methods so far are now suitable for use in the training of supervised algorithms that could predict new formations of anti-HLA antibodies, both DSAs and non-DSAs, after a sensitization event. Confirmation of the prediction of new antibody specificities will validate our approach and will provide the basis of creation of a tool for the optimal selection of donors, especially for highly sensitized patients. Evidently, it would be useful to include samples originating from different populations in order to examine the effects of alleles rarely met in our studied population.

## 5. Conclusions

Overall, keeping in mind that eplet matching appears to be of increased significance for transplantation outcomes, we suggest an identification method of epitopes that mainly drives anti-HLA class II alloreactions based on an agnostic dimensionality reduction approach. In the present study, we determined a limited number of epitopes within each HLA class II locus, which we suggest are the main immunogenic stimulus for around 80% of the recorded responses in the studied population, as shown by PCAs, and could be responsible for the cross reactions observed, such as the development of non-DSAs after solid organ transplantation. We therefore argue that epitope-matching research should focus on this limited number of epitopes that appear to best explain the variance in the Greek population.

## Figures and Tables

**Table 1:** Epitopes found exclusively in each of the groups resulted from PCA and phylogenetic analysis of anti-HLA-DR allele responses. The three distinct antibody groups as observed in PCA analysis are presented in the first column. Eplets within each group are listed in the second column. Eplets in bold are those present in more than one allele in each group and have an intermediate or high Ellipro Score. The third column shows the location of dendrogram antibody groups that appear to recognize the corresponding eplets. This dendrogram clustering confirms most of the immunogenic eplets under discussion. Eplets with very low or low ElliPro scores are considered less likely to drive the respective immune response.

Anti-HLA-DRB Group Responses in PCA	HLA-DR Eplets Recognized by Antibody Groups in PCA	Location of Dendrogram Antibody Groups that Appear to Recognize the Corresponding Eplets	HLA-DR Alleles where the Eplets are Expressed	ElliPro Score
		*Group C*		
*01:01, *01:02, *01:03, *09:01, *09:02, *10:01, *15:01, *15:02, *15:03, *16:01, *16:02, DRB5*01:01 DRB5*02:02	6C	upper	DRB5*02:02	High
	13FE	upper	DRB1*01:01, 01:02, 01:03 DRB1*09:01, 09:02	Very Low
	28H	upper	DRB1*09:01, 09:02 DRB5*01:01 DRB5*02:02	Low
	**30C**	upper	DRB1*01:01, 01:02, 01:03	Intermediate
	30D	upper	DRB5*01:01	Intermediate
	**30G**	upper	DRB1*09:01, 09:02 DRB5*02:02	Intermediate
	30RV	upper	DRB1*10	Intermediate
	**37S**	both	DRB1*01:01, 01:02, 01:03 DRB1*15:01, 15:02, 15:03 DRB1*16:01, 16:02	High
	**71A**	both	DRB1*15:01, 15:02, 15:03 DRB5*02:02	Intermediate
	**96EV**	upper	DRB1*01:01, 01:02, 01:03 DRB5*01:01 DRB5*02:02	High
	**108T**	upper	DRB5*01:01 DRB5*02:02	High
	**142M**	lower	DRB1*15:01, 15:02, 15:03 DRB1*16:01, 16:02	High
		*Group B*		
*04:01 *04:02 *04:03 *04:04 *04:05 *07:01 DRB4*01:01 DRB4*01:03	25Q	left	DRB1*07:01	Intermediate
	**48Q**	left	DRB4*01:01, 01:03	High
	**96Y**	right	DRB1*04:01, 04:02, 04:03, 04:04, 04:05	High
		*Group A*		
*03:01 *03:02 *08:01 *11:01 *11:04 *12:01 *12:02 *13:01 *13:03 *14:01 *14:02 *14:54 DRB3*01:01 DRB3*02:02 DRB3*03:01	**11STS**	upper	DRB1*03:01, 03:02 DRB1*11:01, 11:04 DRB1*13:01, 13:03 DRB1*14:01, 14:02, 14:54	Intermediate
	13SE	both	DRB1*03:01, 03:02 DRB1*11:01, 11:04 DRB1*13:01, 13:03 DRB1*14:01, 14:02, 14:54 DRB3*01:01 DRB3*02:02 DRB3*03:01	Very low
	16Y	both	DRB1*08:01, DRB1*12:01, 12:02	Low
	**31FH**	both	DRB1*03:01, 03:02 DRB1*12:01, 12:02 DRB1*13:01 DRB1*14:01, 14:02, 14:54 DRB3*01:01 DRB3*02:02 DRB3*03:01	High
	37FL	lower	DRB3*01:01	High
	**37L**	lower	DRB1*12:01, 12:02	High
	51R	lower	DRB3*02:02	High
	**57A**	lower	DRB1*14:01, 14:54	High
	**57DE**	lower	DRB1*11:01, 11:04	High
	**70QQ**	both	DRB3*02:02 DRB3*03:01	High
	74L	upper	DRB1*08:01	High
	**74R**	both	DRB1*03:01, 03:02 DRB3*01:01	High
	**77N**	both	DRB1*03:01, 03:02 DRB3*01:01 DRB3*02:02 DRB3*03:01	High
	**96HK**	both	DRB1*03:01, 03:02 DRB1*08:01 DRB1*11:01, 11:04 DRB1*12:01, 12:02 DRB1*13:01 DRB1*14:01, 14:02, 14:54	
	**98Q**	both	DRB3*01:01 DRB3*02:02 DRB3*03:01	Intermediate
	112Y	upper	DRB1*14:01	High
	149H	both	DRB1*03:01, 03:02 DRB1*08:01 DRB1*11:01, 11:04 DRB1*12:01, 12:02 DRB1*13:01 DRB1*14:01, 14:02, 14:54 DRB3*03:01	Very low

**Table 2:** Epitopes found exclusively in each of the groups resulted from 1st–2nd and 2nd–3rd plots of PCA and phylogenetic analysis of anti-HLA-DQ responses. It should be noted that the same groups of antibody responses are formed in PCA and the dendrogram. Eplets within each group are listed in the second column. Eplets in bold are those present in more than one allele in each group and have an intermediate or a high Ellipro score. Eplets with very low or low ElliPro scores are considered less likely to drive the respective immune response. The alloresponse appears to be directed against epitopes predominantly expressed on the DQB1allele chain. DQB1 alleles are indicated in italics.

	HLA-DQ Eplets Recognized by Antibody Groups in PCA	HLA-DQ Alleles where the Eplets are Expressed	ElliPro Score
DQA1*01:01, DQB1*05:01 DQA1*01:01, DQB1*06:02 DQA1*01:02, DQB1*05:02 DQA1*01:02, DQB1*06:02 DQA1*01:02, DQB1*06:04 DQA1*01:02, DQB1*06:09 DQA1*01:03, DQB1*06:01 DQA1*01:03, DQB1*06:03	3P	DQB1*06:01	High
	**30H**	DQB1*05:01, 05:02 DQB1*06:04, 06:03	Intermediate
	**37YV**	DQB1*05:01, 05:02	High
	**52PQ**	*Whole DQB1 group*	High
	**55RPD**	DQB1*06:01, 06:02, 06:03	High
	56PS	DQB1*05:02	High
	**57V**	DQB1*05:01 DQB1*06:04, 06:09	High
	**67VG**	DQB1*05:01, 05:02 DQB1*06:02, 06:03	High
	**70GT**	DQB1*06:02, 06:03	High
	**86A**	DQB1*05:01, 05:02 DQB1*06:01, 06:02, 06:03	Intermediate
	**87F**	DQB1*06:01, 06:02, 06:03	High
	**87Y**	DQB1*05:01, 05:02 DQB1*06:04, 06:09	Intermediate
	**116I**	DQB1*05:01, 05:02	High
	125G	DQB1*06:01, 06:02, 06:03, 06:04, *06:09*	Very low
	125SQ	DQB1*05:01	Low
	**130Q**	DQB1*06:04, 06:09	Intermediate
	**52SK**	Whole DQA1 group	High
	**129QS**	DQA1*01:01, 01:02	High
	130A	DQA1*01:03	High
DQA1*02:01, DQB1*04:01 DQA1*02:01, DQB1*04:02 DQA1*03:03, DQB1*04:01 DQA1*04:01, DQB1*04:02	23L	DQB1*04:01	High
	**56L**	*Whole DQB1 group*	High
DQA1*02:01, DQB1*03:01 DQA1*02:01, DQB1*03:02 DQA1*02:01, DQB1*03:03 DQA1*03:01, DQB1*03:01 DQA1*03:01, DQB1*03:02 DQA1*03:01, DQB1*03:03 DQA1*03:02, DQB1*03:02 DQA1*03:02, DQB1*03:03 DQA1*05:03, DQB1*03:01 DQA1*05:05, DQB1*03:01 DQA1*06:01, DQB1*03:01	45EV	DQB1*03:01	High
	**55PP**	*Whole DQB1 group*	High
	55PPA	DQB1*03:02	High
	55PPD	DQB1*03:01, 03:03	High
	160S	DQA1*05:03	High
DQA1*02:01, DQB1*02:01 DQA1*02:01, DQB1*02:02 DQA1*03:01, DQB1*02:01 DQA1*04:01, DQB1*02:01 DQA1*05:01, DQB1*02:01	**52LL**	*Whole DQB1 group*	High
	135G	DQB1*02:02	High

**Table 3:** Epitopes found exclusively in each of the groups resulted from 1st–2nd plot of PCA and phylogenetic analysis of anti-HLA-DP responses. Eplets within each group are listed in the second column. Eplets in bold are those present in more than one allele in each group and have intermediate or high Ellipro scores. The third column shows the secondary branch of the corresponding dendrogram in which each distinct response is found. This secondary clustering indicates that some of the epitopes might diverge the anti-HLA response to one direction or both. Eplets with very low or low ElliPro scores are less likely to drive the respective immune response.

	HLA-DP Eplets Recognized by Antibody Groups in PCA	Location of Dendrogram Antibody Groups that Appear to Recognize the Corresponding Eplets	HLA-DP Alleles where the Eplets are Expressed	ElliPro Score
DPA1*01:03, DPB1*02:01 DPA1*01:03, DPB1*04:02 DPA1*01:03, DPB1*28:01 DPA1*01:04, DPB1*18:01 DPA1*01:05, DPB1*18:01 DPA1*01:05, DPB1*28:01 DPA1*01:03, DPB1*04:01 DPA1*01:03, DPB1*23:01	**84GGPM**	Upper	DPB1*02:01 DPB1*04:01 DPB1*04:02 DPB1*23:01	High
	178M	Upper	DPB1*04:02	High
DPA1*01:03, DPB1*01:01 DPA1*01:03, DPB1*03:01 DPA1*01:03, DPB1*06:01 DPA1*01:03, DPB1*11:01 DPA1*01:03, DPB1*19:01 DPA1*01:05, DPB1*03:01 DPA1*02:01, DPB1*01:01 DPA1*02:01, DPB1*03:01 DPA1*02:01, DPB1*05:01 DPA1*02:01, DPB1*06:01 DPA1*02:01, DPB1*09:01 DPA1*02:01, DPB1*10:01 DPA1*02:01, DPB1*13:01 DPA1*02:01, DPB1*14:01 DPA1*02:01, DPB1*15:01* DPA1*02:01, DPB1*18:01* DPA1*02:01, DPB1*17:01 DPA1*02:02, DPB1*05:01 DPA1*02:02, DPB1*10:01 DPA1*02:02, DPB1*11:01 DPA1*02:02, DPB1*13:01 DPA1*03:01, DPB1*13:01 DPA1*03:01, DPB1*20:01 DPA1*04:01, DPB1*28:01*	9H	Left	DPB1*09:01 DPB1*10:01 DPB1*14:01 DPB1*17:01	Low
	9YL	Both	DPB1*03:01 DPB1*06:01 DPB1*11:01 DPB1*13:01 DPB1*20:01	Low
	11L	Both	DPB1*03:01 DPB1*06:01 DPB1*09:01 DPB1*10:01 DPB1*11:01 DPB1*13:01 DPB1*14:01 DPB1*17:01 DPB1*20:01	Very Low
	**33EYA**	Right	DPB1*01:01 DPB1*13:01	High
	35LV	Right	DPB1*05:01	High
	**35YA**	Right	DPB1*01:01 DPB1*11:01 DPB1*13:01 DPB1*15:01	High
	**55EAE**	Right	DPB1*05:01 DPB1*19:01	High
	**57D**	Left	DPB1*03:01 DPB1*06:01 DPB1*09:01 DPB1*14:01 DPB1*17:01 DPB1*20:01	High
	65LE	Left	DPB1*06:01	High
	**69R**	Right	DPB1*11:01 DPB1*15:01	High
	**76I**	Right	DPB1*13:01 DPB1*19:01	Intermediate
	**76V**	Both	DPB1*01:01 DPB1*03:01 DPB1*09:01 DPB1*10:01 DPB1*14:01	Intermediate
	**84DEAV**	Both	DPB1*01:01 DPB1*03:01 DPB1*05:01 DPB1*06:01 DPB1*09:01 DPB1*10:01 DPB1*11:01 DPB1*13:01 DPB1*14:01 DPB1*17:01 DPB1*20:01	High
	11M	Both	DPA1*02:02 DPA1*03:01	Very low
	31Q	Both	DPA1*02:01 DPA1*02:02	Very low
	**50R**	Both	DPA1*02:01 DPA1*02:02 DPA1*04:01	High
	66S	Both	DPA1*03:01	High
	111R	Both	DPA1*02:01 DPA1*02:02	Low
	**127P**	Both	DPA1*02:01 DPA1*02:02 DPA1*04:01	High
	**160V**	Both	DPA1*02:01 DPA1*02:02 DPA1*04:01	High
	190A	Both	DPA1*04:01	High

## Data Availability

Data are available from the authors by request.

## Supplementary Materials

The following supporting information can be downloaded at: https://www.mdpi.com/article/10.3390/jcm11237046/s1, Figure S1: Scree plot depicting contribution of each principal component to total variance explained. Figure S2: PCA biplot projections of anti-HLA-DR responses. Projection of the third and fourth eigenvectors of the covariance matrix. Each arrow represents a specific anti-HLA-DR reaction, while each point indicates individual reactions. Color of loadings and points represent the cos2 value of the explained variance for variables and individuals respectively.
