# Supplementary material for: An Approach to Identify HLA Class II Immunogenic Epitopes in the Greek Population through Machine Learning Algorithms
Source: J Clin Med. 2022 Nov 29;11(23):7046. doi: 10.3390/jcm11237046 (PMC9738260; doi:10.3390/jcm11237046)
Supplement: Supplementary file 1 [file jcm-11-07046-s001.zip › jcm-2016513-supplementary.pdf]

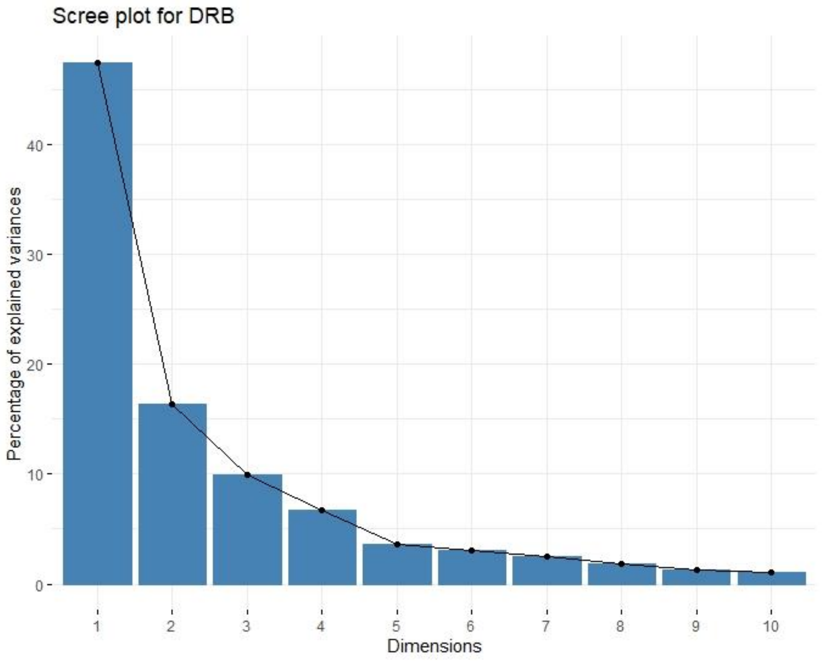

Supplementary Figure S1. Scree plot depicting contribution of each principal component to total variance explained.

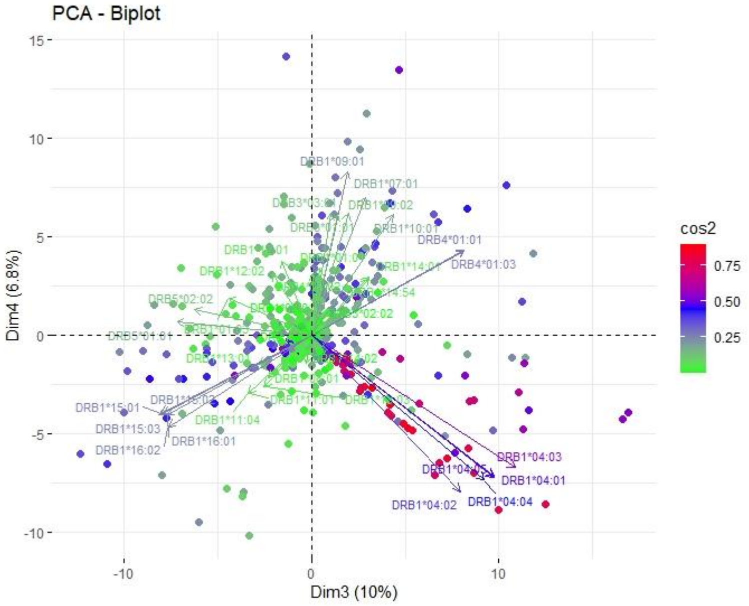

Supplementary Figure S2. PCA biplot projections of anti-HLA-DR responses. Projection of the third and fourth eigenvectors of the covariance matrix. Each arrow represents a specific anti-HLA-DR reaction, while each point indicates individual reactions. Color of loadings and points represent the cos2 value of the explained variance for variables and individuals respectively.
